# Minimizing detection bias of somatic mutations in a highly heterozygous oak genome

**DOI:** 10.1093/g3journal/jkaf143

**Published:** 2025-06-21

**Authors:** Wenfei Xian, Pablo Carbonell-Bejerano, Fernando A Rabanal, Ilja Bezrukov, Philippe Reymond, Detlef Weigel

**Affiliations:** Department of Molecular Biology, Max Planck Institute for Biology Tübingen, Tübingen 72076, Germany; Department of Molecular Biology, Max Planck Institute for Biology Tübingen, Tübingen 72076, Germany; Instituto de Ciencias de la Vid y del Vino, ICVV, CSIC, Universidad de La Rioja, Gobierno de La Rioja, Logroño, La Rioja 26007, Spain; Department of Molecular Biology, Max Planck Institute for Biology Tübingen, Tübingen 72076, Germany; Department of Molecular Biology, Max Planck Institute for Biology Tübingen, Tübingen 72076, Germany; Department of Plant Molecular Biology, University of Lausanne, Lausanne 1015, Switzerland; Department of Molecular Biology, Max Planck Institute for Biology Tübingen, Tübingen 72076, Germany; Institute for Bioinformatics and Medical Informatics, University of Tübingen, Tübingen 72076, Germany

**Keywords:** Somatic mutations, *Quercus robur*, HiFi assembly

## Abstract

Somatic mutations are particularly relevant for long-lived organisms. Sources of somatic mutations include imperfect DNA repair, replication errors, and exogenous damage such as ultraviolet radiation. A previous study estimated a surprisingly low number of somatic mutations in a 234-year-old individual of the pedunculate oak (*Quercus robur*), known as the Napoleon Oak. It has been suggested that the true number of somatic mutations was underestimated due to gaps in the reference genome and too conservative filtering of potential mutations. We therefore generated new high-fidelity long-read data for the Napoleon Oak (*n* = 12) to produce both a pseudo-haploid genome assembly and a partially phased diploid assembly. The high heterozygosity allowed for complete reconstruction of phased and gapless centromeres for 22 of the 24 chromosomes. On the other hand, the high heterozygosity posed challenges for short-read alignments. Use of only the pseudo-haploid assembly as a reference led to potential misalignments, while use of only the diploid assembly reduced variant detection sensitivity. Since most somatic mutations are layer-specific, the fraction of reads covering a specific somatic mutation is expected to be relatively low, even where all cells in a single layer contain a specific mutation. To address this challenge, we employed a read assignment strategy, selecting the appropriate reference sequence (pseudo-haploid or diploid) based on alignment score and mapping quality. Ultimately, we identified 198 high-confidence somatic mutations, compared with 17 somatic mutations identified before with the same set of short reads. Our approach thus increased the total estimated annual mutation rate by a factor of 5.

## Introduction

Fifteen years ago, a seminal study by Sally Otto and colleagues showed that mutations reducing male fertility accumulate in a long-lived deciduous tree in a clock-like manner, suggesting that there are natural limits to how long trees could produce viable offspring (Ally *et al.* 2010). Since that early paper, a series of studies has attempted to directly detect the accumulation of mutations in trees using a range of sequencing strategies, resulting in a remarkably wide range of mutation rate estimates (summarized in Johannes 2024).

Oaks, widely distributed in Northern Hemisphere forests, are renowned for their longevity, with many individuals living for centuries (Leroy *et al.* 2020). One noteworthy, over 200-year-old specimen, known as the Napoleon Oak, which grows on the campus of the University of Lausanne, was the subject of a recent somatic mutation study (Schmid-Siegert *et al.* 2017), which used a reference genome assembled from long reads of this individual in combination with short reads of material from a lower and an upper branch. Ten single nucleotide variants (SNVs) were identified in the upper branch and 7 SNVs in the lower branch. Taking into account potential false negatives, the fixed somatic mutation rate was estimated to be 4.2–5.2 × 10⁻⁸ substitutions per site per generation (Schmid-Siegert *et al.* 2017). Subsequent work with another oak tree suggested that the number of somatic mutations identified in the Napoleon Oak was a likely underestimate because the minimization of false positives (specificity) had been prioritized over the identification of all true positives (sensitivity) (Plomion *et al.* 2018). Apart from the reference genome having been generated with an early long-read technology, the threshold for variant calling might have been overly stringent. In addition, the high heterozygosity of oak genomes could have impacted variant calling, as only a single phase of the Napoleon Oak genome had been assembled.

When reads are aligned to a haploid assembly, regions that are highly divergent in sequence between the 2 haplotypes might produce misalignments, as a haploid reference cannot fully represent both haplotypes. This has been quantitatively confirmed in highly heterozygous sweet oranges, where a phased assembly as reference yielded more than twice the number of somatic mutations compared to a haploid assembly (Wang *et al.* 2024). Using a diploid reference is, however, not without its own challenges. In regions that are identical in the 2 haplotypes, aligners will randomly assign reads to 1 of the 2 phases, reducing the power of variant identification, particularly when there are only very few variant reads covering a specific position. Conversely, high-frequency variants may appear on both haplotypes, leading to an overestimate of true variants.

A recent study of 2 tropical trees found that almost all somatic mutations are present at low frequency in the sampled tissue (Schmitt *et al.* 2024). This finding highlights the difficulty of distinguishing somatic mutations that are not present in all cells of the sampled tissue from sequencing errors. This is a particular issue for plants, where multiple tissue layers are established early on in development, with these layers staying largely segregated throughout the life of the plant (Steeves and Sussex 1989). Thus, even somatic mutations that are present in all cells of a layer in the sampled tissue may go undetected if allele frequency thresholds are applied too strictly. Several studies have confirmed that most somatic mutations are layer-specific, as has long been known from the study of mutations with phenotypic impact, such as grape color (Dermen 1960; Amundson *et al*. 2023; Goel *et al.* 2024). Since the samples sequenced in the Napoleon Oak study were the entire leaves (Schmid-Siegert *et al.* 2017), this might have further reduced the observed frequency of detected somatic mutations. These considerations underscore the need for more sensitive alignment and variant-calling strategies that can accurately capture true mutations, even when present at low frequencies.

We produced a new genome assembly of the Napoleon Oak and optimized a somatic mutation-calling pipeline for highly heterozygous genomes, using both haploid and diploid reference genomes. Utilizing this improved approach, we identify a larger set of high-confidence somatic mutations in the Napoleon Oak, which lead to a substantial upward revision of the annual somatic mutation rate.

## Methods

### Biological material and PacBio HiFi sequencing

Six grams of leaves were sampled on August 2021 from the bottom branch of the Napoleon Oak (*Quercus robur*) on the campus of the University of Lausanne (Switzerland, 46° 31′ 18.9″ N, 6° 34′ 44.5″ E). Leaves were stored in liquid nitrogen. Prior to DNA extraction, to avoid contamination from pathogens, we removed any visibly spotted areas of the leaves. To improve DNA extraction efficiency, we also trimmed away the leaf veins. High molecular weight (HMW) DNA was obtained from frozen leaf powder as described by Calderón *et al.* (2024). In brief, the Nanobind Plant Nuclei Big DNA Kit (Circulomics) was used to extract HMW DNA from nuclei isolated according to Workman *et al.* (2018). We sheared the HMW DNA into appropriately sized fragments using a g-TUBE and then prepared a PacBio library using the SMRTbell Express Template Prep Kit 2.0 from 10 µg of sheared DNA. The library was selected for >10 kb fragments using a BluePippin instrument (Sage Science). Sequencing was performed using two 8 M SMRTCells on a Sequel II.

### Genome assembly and annotation

PacBio CCS (https://github.com/PacificBiosciences/ccs) (v6.4.0) was used to generate CCS reads from the raw subreads with the parameter --min-rq=0.88. Subreads were aligned to CCS reads with ACTC (https://github.com/PacificBiosciences/actc) (v0.3.1). CCS reads were further polished by DeepConsensus (Baid *et al*. 2023) (v1.2.0) resulting in 63.65 Gb high-quality HiFi reads. Short and long reads of sample 0 were used to estimate the heterozygosity from kmer frequencies using KMC3 and GenomeScope v2.0.

HiFi reads were assembled into a haploid assembly using Hifiasm v0.24.0 (Cheng *et al.* 2021), producing 16 long contigs. Based on length ranking, the 16th contig was 3.5 Mb long, while the 17th contig was much smaller, only 0.5 Mb. Therefore, we retained only the 16 longer contigs. Among these, 8 contained telomeric repeats at both ends, and the remaining 8 contigs had telomeric repeats at only 1 end. A diploid assembly was produced with Verkko v1.4.1 (Rautiainen *et al.* 2023) with default parameters.

Commonly used scaffolding methods rely on reference genomes (Alonge *et al.* 2022), but this approach may introduce reference bias. Moreover, only 4 gaps remained in our haploid assembly, 3 of which likely contained rDNA clusters. Previous studies indicated that rDNA clusters form tangles in the Verkko assembly graph, and we visualized the Verkko diploid assembly graph (Rautiainen *et al.* 2023) assembly with Bandage v0.9 (Wick *et al.* 2015) to identify tangles caused by rDNA clusters. We manually selected the node IDs corresponding to each tangle in Bandage—representing 4 haplotype-phased 45S rDNA clusters and 1 haplotype-collapsed 5S rDNA cluster—and used them as input for Ribotin to generate consensus sequences. The 18S, 5.8S, and 25S rDNA units from *Arabidopsis thaliana* were aligned to each 45S rDNA consensus sequence, and the 5S rDNA unit from *A. thaliana* was aligned to the oak 5S rDNA consensus sequence using both BWA 0.7.17-r1188 (Li and Durbin 2009) and BLASTN 2.12.0+ (Camacho *et al.* 2009). A flow diagram of the described assembly steps is shown in Supplementary Fig. 14.

Using Minimap2 2.24-r1122 (Li 2018), we aligned the 8 contigs with telomeric repeats at only 1 end to the Verkko assembly. From the unitig-popped.layout.scfmap file, we identified the corresponding nodes in the assembly graph. Based on the connectivity information in the graph, we manually linked 2 contigs with 100 Ns, ultimately obtaining 12 pseudo-chromosomes. Chromosomes were assigned to these 12 scaffolds based on alignment to the 3P Oak genome assembly (Plomion *et al.* 2018). Therefore, the haploid assembly refers to the scaffolded sequences, while the diploid assembly corresponds to the Verkko contigs. We assembled the oak organellar genome using TIPPo v2.1 (Xian *et al.* 2025).

Quality evaluation of our haploid assembly with Merqury v1.3 indicated a QV of 53 (Rhie *et al.* 2020) with short reads only, and Compleasm assessment showed 99% completeness of the embryophyta_odb10 conserved gene set (Huang and Li 2023). The same evaluations were also performed on the previous assembly of the Napoleon Oak (Schmid-Siegert *et al.* 2017) for comparison.

Liftoff (Shumate and Salzberg 2021) was used to project protein-coding genes from the 3P Oak assembly (Plomion *et al.* 2018) onto the Napoleon Oak assembly. Transposable elements were annotated with EDTA v2.0.0 (Ou *et al.* 2019) with parameters --sensitive 1 --anno 1. CpG methylation profiles were identified from HiFi reads by ccsmeth (Ni *et al.* 2023). Satellite repeats were annotated with Tandem Repeat Annotation and Structural Hierarchy (TRASH) (Wlodzimierz *et al.* 2023).

To verify the accuracy of the CEN146 array, HiFi reads were aligned to the diploid assembly with Minimap2 (Li 2018), Winnowmap2 (Jain *et al.* 2022), and VerityMap (Bzikadze *et al.* 2022). StainedGlass (Vollger *et al.* 2022) and pyGenomeTracks (Lopez-Delisle *et al.* 2021) were used to visualize HiFi read coverage, TE annotation, and the similarity of the CEN146 arrays.

CEN146 arrays from homologous chromosomes were aligned separately using UniAligner (Bzikadze and Pevzner 2023) and Minimap2 (Li 2018). Alignment length and mismatch count were analyzed using UniAligner's built-in script, cigar_histogram.py. Dot plots of rare kmers were generated using smart_dotplot.py, also from UniAligner. For Minimap2 alignments, dot plot visualization was performed using paf2dot (https://github.com/pangenome/paf2dot). To select unique homologous chromosomes, we manually identified 2 contigs forming a bubble in the assembly graph and aligned them using Minimap2. The specific sequences can be found in Supplementary Table 7.

### Short-read processing and alignment

The Illumina short reads for the upper and lower branches have been published (Schmid-Siegert *et al.* 2017) and were downloaded from NCBI (accession PRJNA327502). Reads were trimmed using fastp v0.23.1 (Chen *et al.* 2018) with the parameters -q 20 -l 75 -w 12 --cut_tail --cut_mean_quality 20.

Trimmed reads were separately aligned to the diploid and haploid assemblies using BWA 0.7.17-r1188 (Li and Durbin 2009). Potential PCR duplicates were marked using Picard v2.26.7 (“Picard toolkit” 2019).

### Alignment comparison between haploid and diploid assemblies as reference

We compared insert size distribution, pairing orientation, and the proportion of properly paired reads between haploid and diploid assemblies as reference using samtools v1.10 stat.

To identify the potential misalignment of short reads, we first aligned the short reads to the diploid assembly to serve as a reference framework and then extracted the flanking 2 kb sequences for every read and aligned them to the haploid assembly with Minimap2 2.24-r1122. Using the flanking sequences from the diploid assembly as an intermediary, we inferred the expected positions of short reads in the haploid assembly and compared these to the actual alignment positions when short reads were mapped directly against the haploid genome. Reads that did not align within the range of expected positions were identified (Supplementary Fig. 10).

We used DeepVariant v1.8.0 (Poplin *et al*. 2018) to call germline SNVs on alignments of short reads from either the upper or lower branch to the haploid assembly. To ensure the reliability of variants identified in the short-read dataset, we retained only variants with a depth between 0.5× and 2× sample coverage, a minimum variant allele frequency (VAF) of 0.3, and those marked as PASS. Because DeepVariant may produce false negatives, we extracted the variants identified in the short-read data and used Bam-readcount v1.0.1 (Khanna *et al*. 2022) to verify whether these variants were present in the HiFi data. We applied a very lenient threshold: if a variant was supported by at least 5 HiFi reads (the genome-wide coverage was 77×), we considered it reproduced in HiFi data.

### Identification of somatic mutations

We extracted the MAPQ and alignment scores for each read from the BAM files of short reads aligned to either the haploid or diploid reference genome. If the alignment scores differed, a read was assigned to the reference genome with the higher alignment score. If the alignment scores were the same, the read was assigned to the reference genome with the higher MAPQ value. If the MAPQ values were also identical, the read was assigned to the diploid assembly. Clipped reads were removed. Reads from the upper and lower branches were used as the mutant and control sample, respectively.

For the diploid assembly as the reference genome, we used bam-readcount v1.0.1 (Khanna *et al*. 2022) with MAPQ of ≥0 and base quality of ≥0 for the control sample. These lenient thresholds were used to reduce false negatives from the control. Only references called sites with coverage of ≥20×, and no indels, were collected as the “set of control positions”.

For the mutant sample, to reduce false positives, we applied stringent parameters using bam-readcount v1.0.1 (Khanna *et al*. 2022): MAPQ of ≥10, base quality of ≥30, and site coverage of ≤70×. Sites had to have biallelic bases without indels. The alternate allele had to have at least 3 supporting reads, with support on both strands. Reads with the reference allele had to have an average mismatch of ≤0.01. The average mismatch of reads carrying the alternative allele minus the mismatch of reads carrying the reference allele had to be ≤0.01. Only variant sites in the mutant sample overlapping with the “set of control positions” identified in the control sample were retained as likely somatic mutations.

For the haploid assembly as the reference genome, we used a similar workflow for the diploid assembly as reference, but with the following adjustments: control sample, coverage of >40×, mutant sample, and coverage of ≤120×. The somatic mutations identified against both the haploid and diploid assemblies were then combined. For comparison, Strelka2 v2.9.10 (Kim *et al.* 2018) was used to detect the somatic mutations using separately either the haploid or diploid assembly.

In the diploid assembly, we require a minimum depth of 20× for the control sample. We then quantified regions in the diploid assembly where the sequencing depth is above 20×, resulting in an effective site size of 1.3 Gb.

To identify the position of previously validated SNVs in the new assemblies, we extracted the flanking 100 bp from the previous Napoleon Oak assembly (Schmid-Siegert *et al.* 2017) and aligned the sequences to both our haploid and diploid assemblies with Minimap2 (Li 2018). To calculate the allele frequency of the 9 somatic SNVs shared between leaves and acorns in the 3P Oak (Plomion *et al.* 2018), we downloaded short reads from NCBI (accession number PRJEB8388). We used the same approach as for the Napoleon Oak to process and align the reads to the assembly. We extracted the allele frequency of the 9 SNVs from the bam file.

## Results

### Genome architecture of *Quercus robur*

**Fig. 1.**
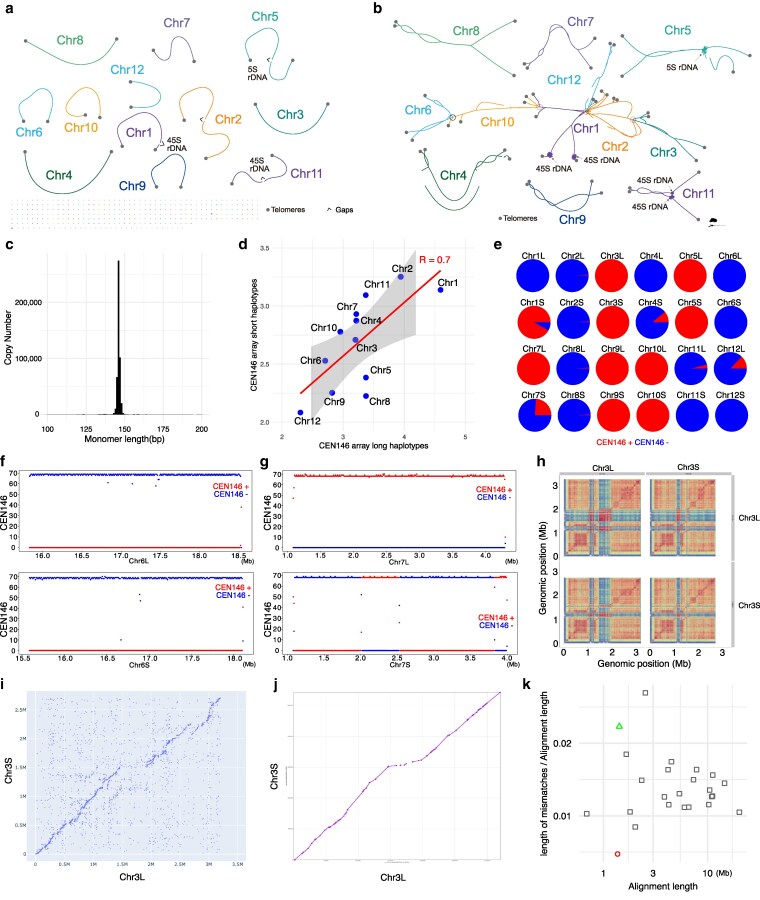
Genome architecture of the Napoleon Oak. a) Scaffolded haploid assembly graph. b) Contig-level diploid assembly graph. c) Copy number of centromeric satellite repeats in the diploid assembly. d) Length of CEN146 arrays on different chromosomes. e) Orientation distribution of CEN146: forward (red, +) and reverse (blue, −) proportion for each chromosome and haplotype (homolog). L, longer haplotype; S, shorter haplotype. f) Strand-specific density of CEN146 in 10-kb windows along the CEN146 arrays on the 2 chromosome 6 haplotypes. Forward strand density in red and reverse strand density in blue. g) Strand-specific density of CEN146 in 10-kb windows along the CEN146 arrays on the 2 chromosome 7 haplotypes. Forward strand density in red and reverse strand density in blue. h) Heat maps depicting sequence identity within and between the 2 haplotypes of the CEN146 array on chromosome 3. i) Dot plot of rare kmers showing that the 2 haplotypes of the CEN146 array on chromosome 3 can be aligned with UniAligner. j) Minimap2 alignment of the CEN146 array on chromosome 3. k) Apparent substitution rates across various regions: green triangle represents chromosome 3 CEN146 arrays aligned using Minimap2, red circle indicates alignments using UniAligner, and gray rectangles indicate unique non-CEN146 sequences aligned with Minimap2. For details of the unique regions, see Supplementary Table 7.

To identify high-quality somatic mutations in the diploid pedunculate oak (*Q. robur*, *n* = 12), individually known as the Napoleon Oak, we first assembled a high-quality genome of this individual. In August 2021, fresh leaves were sampled from the bottom branch, at the same location where the material used for genome assembly and mutation calling had been sampled before (sample 0 of Schmid-Siegert *et al.* 2017). Two PacBio SMRTcells were used to generate a total of 63.7 Gb of HiFi data (Supplementary Table 1). Using Hifiasm (Cheng *et al.* 2021), we assembled a highly contiguous haploid genome comprising only 16 contigs (Fig. 1a). Eight of the contigs contained telomeric repeats at both ends and thus corresponded to 8 complete chromosomes.

We estimated a high level of heterozygosity, about 1.6%, for the Napoleon Oak by kmer-based analysis of Illumina short and PacBio long reads (Supplementary Fig. 1). Because the haploid Hifiasm assembly only partially represents all of the sequences present in the genome, we assembled a diploid genome from the same HiFi data using Verkko (Rautiainen *et al.* 2023; Fig. 1b).

We used information from the Verkko assembly graph to scaffold the 8 contigs of the Hifiasm assembly that did not have telomeres on both ends into additional chromosomes (Supplementary Table 2). One of the major challenges in assembling complete plant chromosomes comes from rDNA clusters (Rabanal *et al.* 2022; Fultz *et al.* 2023), and the genome of the pedunculate oak contains two 45S rDNA clusters and one 5S rDNA cluster (Bočkor *et al.* 2014). In the Verkko assembly graph, there were prominent tangles that connected sequences at both ends. Based on published descriptions (Rautiainen *et al.* 2023), we expected these tangles to correspond to rDNA clusters. We derived consensus sequences of each rDNA clusters and found these to have high similarity (>95%) to those of *A. thaliana*, although the lengths of the 45S rDNA units differed between chromosome 1 (9.8 kb) and chromosome 11 (7.7 kb) (Supplementary Table 15 and Supplementary Fig. 13).

We aligned the 8 contigs of the Hifiasm assembly without telomeres on both ends to the diploid assembly and projected them onto the diploid assembly graph (Supplementary Fig. 2). Based on the connections in the graph, we manually scaffolded the 8 contigs, resulting in 4 additional chromosome-level pseudomolecules. Each had 1 end capped by telomere repeats and large arrays of tandem repeats (either rDNA or another, unknown repeat type) at the other end (Supplementary Table 2). The final haploid genome had high accuracy (QV 53) and high completeness (BUSCO 99.8%), significantly surpassing the quality of the previous version generated from an earlier generation of long reads (Supplementary Fig. 3 and Supplementary Table 3) (Schmid-Siegert *et al.* 2017). The assembled haploid genome spans 810 Mb, in agreement with estimates for the 1C genome size for this species, which range from 759 to 1,068 Mb (Pellicer and Leitch 2020).

The long and highly accurate PacBio HiFi reads enabled the assembly of repetitive regions, such as centromeres (Naish *et al.* 2021). In each of the 12 chromosomes of the haploid assembly, we identified large arrays of a shared 146 bp satellite repeat unit, with a genome-wide total of 486,051 copies in the diploid assembly (Fig. 1c; Supplementary Fig. 4 and Supplementary Table 4) and with high levels of CpG methylation (Supplementary Fig. 5). These arrays, on average about 3 Mb long, are likely centromeres, with 22 of 24 being gapless and phased. In analogy with the *A. thaliana* nomenclature, we call these repeats CEN146 (Naish *et al.* 2021). The CEN146 arrays on chromosome 2 were manually scaffolded (Supplementary Table 5). Uniform coverage of HiFi read alignments suggest that there are no significant assembly errors (Supplementary Fig. 6). Within the CEN146 arrays, transposons were rare, but transposons were present in the flanking regions (Supplementary Fig. 6).

For each set of homologs, we categorized the CEN146 arrays as belonging to a long haplotype (L) or short haplotype (S) based on their lengths. The size of CEN146 arrays differed more between chromosomes than between homologs (Fig. 1d). The relative orientation of CEN146 repeats within the arrays was homolog (haplotype) rather than chromosome-specific (Fig. 1e–g). For instance, on Chr1S, Chr4S, Chr7S, and Chr12L, more than 8% of repeat units were in an orientation opposite to the majority of repeats, whereas in their counterparts (Chr1L, Chr4L, Chr7L, and Chr12S), over 99% of arrays were in the same orientation.

Due to the challenges of aligning long highly repeated sequences, previous studies have often greatly differed in their estimates for evolutionary turnover of centromere sequences (Logsdon *et al.* 2021; Bzikadze and Pevzner 2023). Here, we attempted to use our phased CEN146 arrays to examine the performance of 2 different alignment approaches for the discovery of centromeric variants: Minimap2 (Li 2018), based on standard concepts of molecular evolution, and UniAligner (Bzikadze and Pevzner 2023), which is designed for aligning long tandem repeats. First, we used UniAligner to align the 2 haplotypes of the CEN146 arrays of each chromosome. Dot plots of rare kmers revealed that only chromosome 3 contained a substantial number of rare kmers, indicating that alignment was feasible only for chromosome 3 (Fig. 1i and Supplementary Fig. 7). Because only chromosome 3 produced a Minimap2 alignment consistent with the UniAligner dot plot (Fig. 1j and Supplementary Fig. 8), we focused on comparing the CEN146 arrays of this chromosome (Fig. 1h). As UniAligner uses rare kmers as anchors to extend alignments, we used the alignment blocks of Minimap2 with the maximally possible mapping quality (MAPQ) of 60. The total alignment lengths were similar for the 2 tools, with UniAligner aligning 1.36 Mb and Minimap2 aligning 1.41 Mb. However, the substitution rate inferred by UniAligner is 0.004, which is 5 times lower than the one inferred by Minimap2, 0.02. Notably, the substitution rate from Minimap2 was higher than that of most non-CEN146 uniquely aligned regions using Minimap2 (Fig. 1k and Supplementary Tables 6 and 7). Because the 2 tools produced such diverging results, we excluded the centromeres from our analyses of somatic mutation rates.

### Variant allele frequency estimates of layer-specific mutations

Our goal is to identify somatic mutations that had become fixed in stem cells that gave rise to the sampled tissue (Schmid-Siegert *et al.* 2017). We take the cell layer where the mutations occur into account, since the layer specificity of somatic mutations has not only long been known from phenotypic examination (Dermen 1960), subsequently confirmed by molecular analysis of specific mutations (Kobayashi *et al.* 2004), but has also recently shown to be the prevailing type by high-throughput sequencing in potato, apricot, and apple (Amundson *et al*. 2023; Goel *et al.* 2024 ; Sun *et al.* 2024).

The plant body comprises multiple, segregated layers of cells that arise from multiple layers of stem cells in the meristems (Steeves and Sussex 1989), where the outermost layer, L1, makes up the epidermis, L2 the photosynthetic tissue, and L3 the ground tissue. The frequency with which fixed somatic mutations are observed in samples such as leaves, which include cells derived from all meristem layers, will depend on the relative contribution of each layer to the total genomic DNA in that sample. Although we lack layer-specific data to evaluate the DNA content of each layer in oak leaves, one can roughly approximate the DNA content of L2 by analyzing shared mutations detected in leaves and acorns, since embryonic tissues originate from L2 (Amundson *et al*. 2023).

**Fig. 2.**
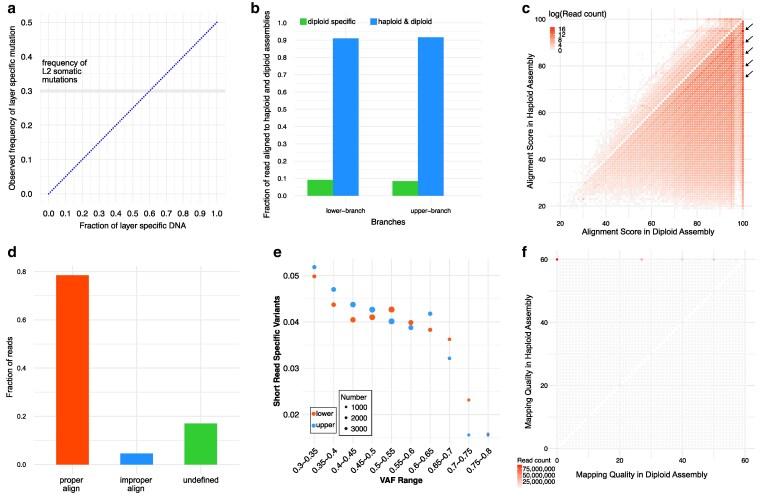
Using haploid or diploid assemblies for short-read alignment. a) Frequencies of somatic mutations in sequencing reads relating to the contribution of a layer to the total DNA of the sample. b) Fractions of reads mapping only to the diploid assembly. c) Distribution of mapping (alignment) scores in 2 assemblies as reference. The dots indicated by arrows represent germline single nucleotide variants. d) Reads whose mapping (alignment) position matched the expected position (as inferred from alignments of 4.1 kb diploid assembly fragments centered on each read to the haploid assembly) were classified as having a proper alignment. Reads that aligned outside the expected position were classified as having an improper alignment. Undefined are reads where the 4.1 kb diploid assembly region centered on the read aligned to a region in the haploid assembly that was smaller than 4 kb. e) Fraction of variants identified with short reads that were not identified from HiFi reads. The sizes of the dots indicate the number of short-read-specific variants. f) Distribution of mapping qualities when using either the haploid or diploid assembly as reference.

In the published oak accession 3P, 9 somatic SNVs were shared between leaves and the embryonic tissue in acorns (Plomion *et al.* 2018). Thus, these 9 SNVs are most likely derived from L2 of the meristem and can be considered as L2 markers. The median frequency of reads covering these 9 somatic mutations in the leaves was approximately 0.3 (Supplementary Table 8). Since a fixed mutation will appear in only 1 haplotype, the DNA of the mutant cells would have constituted 60% of the total DNA of the sample (Fig. 2a). This provides an upper bound for the contribution of the other 2 layers, with L1 contributing less than L3. Given that L1 and L3 together make up about 40% of all reads, this would suggest that L1 contributes <20% and L3 >20%. The expected observed frequency of L1-fixed SNVs in the haploid assembly, which serves as the reference, would thus be below 0.1 (below half of 20%). Therefore, the use of a VAF threshold substantially above 0.1 may prevent the identification of layer-specific somatic mutations even when they are fixed in the investigated sample.

### Limitations of using either a single haploid or diploid assembly as reference

In highly heterozygous genomes, a single haploid assembly is an imperfect representation of the entire genome, and reads from regions that are highly diverged in the other haplophase cannot be aligned. We compared the short-read IDs that mapped to the haploid assembly vs those mapped to the diploid assembly and found that approximately 9% of the reads could only be aligned to the diploid assembly (Fig. 2b; Supplementary Table 11). Therefore, using only the haploid assembly as a reference genome will increase the false-negative rate in mutation identification.

Even though more reads can be mapped to the diploid than to the haploid assembly, the number of paired reads with discordant orientation or mapping to different chromosomes is reduced (Supplementary Table 12). Additionally, when reads are mapped to the diploid assembly, the average size of mapped fragments is smaller, with reduced standard deviation. This suggests that mapping to the diploid assembly can increase read mapping accuracy and reduce false positives.

We extracted reads that mapped to both assemblies and compared their alignment scores in the haploid and diploid assemblies (Fig. 2c and Supplementary Fig. 9). Although a correct alignment generally results in a higher alignment score, when aligning short reads to a haploid genome, heterozygous sites appear as mismatches, with BWA assigning +1 for a match and −4 for a mismatch by default (Li and Durbin 2010). For reads with an alignment score of 100 in the diploid assembly, alignment scores in haploid assembly values are predominantly 95, 90, 85, 80, or 75. Overall, aligning reads to the diploid genome increases the alignment score, indicating a reduction in alignment errors.

Because short reads are more prone to mismapping than long reads, we used long flanking regions from the diploid assembly to identify mismapped short reads in the haploid assembly. This analysis revealed that approximately 5% of reads aligned to the haploid genome were mismapped relative to their expected positions (Fig. 2d and Supplementary Table 14).

To further evaluate potential mapping errors introduced by using the haploid assembly as a reference, we examined whether germline SNVs identified in short-read data could be confirmed in the HiFi reads. As expected, the fraction of short-read-specific variants decreased as the VAF of short-read variants increased. Notably, when the VAF of short-read variants was between 0.3 and 0.35, the proportion of short-read-specific variants remained around 5% (Fig. 2e and Supplementary Table 13).

While aligning reads to a diploid genome can mitigate mapping errors caused by differences between the 2 haplotypes, using only the diploid genome also has limitations. For instance, in homozygous regions, MAPQ is often 0 (due to multiple mapping), and coverage is reduced by half, making low-frequency mutations more difficult to detect. Among reads with different MAPQ values when mapped against either the haploid or diploid reference assembly, most had a MAPQ of 60 in the haploid assembly, but a MAPQ of 0 in the diploid assembly (Fig. 2f).

### Optimizing short-read alignments in highly heterozygous genomes via dynamic reference genome selection

**Fig. 3.**
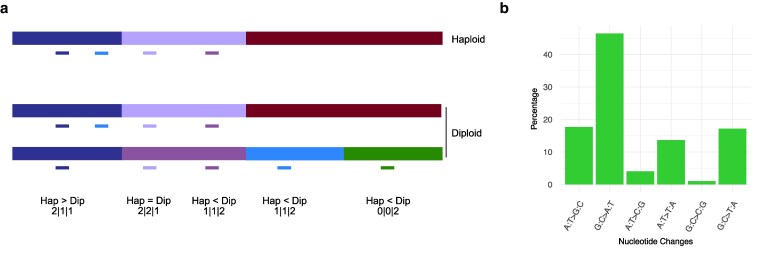
Somatic mutations in the Napoleon Oak. a) Diagram of how inappropriately aligned short reads in the haploid and diploid assembly can be identified. Scores indicate alignment in haploid and in the diploid genome composed of 2 haplophases. 2, high alignment and MAPQ scores; 1, high alignment but low MAPQ scores, or low alignment but high MAPQ scores; 0, no alignment. Dark blue read: homozygous region, reads in both assemblies have the same alignment scores, but the MAPQ value is higher in haploid than in diploid assembly. Light purple read: heterozygous region, read has higher alignment score and MAPQ value in haploid vs diploid assembly, and alignment score differs between haplophases. Dark purple read: heterozygous region, read has higher alignment score in diploid vs haploid assembly, and alignment score differs between haplophases. Light blue read: heterozygous region, read has higher alignment score in diploid vs haploid assembly, and alignment score differs between haplophases. Green read: highly divergent region, read only aligned to 1 haplophase of the diploid assembly. b) Mutation spectrum for all 198 somatic SNVs.

To overcome these issues, we utilized both haploid and diploid assemblies as reference genomes. The haploid assembly was employed to identify variants in regions identical in both haplotypes, while the diploid assembly was leveraged to resolve heterozygous regions unique to each haplotype. We followed a hierarchical decision-making strategy, in which reads were first aligned to both haploid and diploid assemblies and each read was then assigned to 1 of the 2 reference assemblies based on a simple prioritization rule: if a read had a higher alignment score in 1 assembly, it was assigned to that assembly. If alignment scores were identical, the read was assigned to the assembly with the higher MAPQ value. Finally, if both alignment scores and MAPQ values were equal, the read was assigned to the diploid assembly (Fig. 3a). The details of the variant-calling pipeline and subsequent filtering criteria are described in the Methods.

### Somatic mutations in the Napoleon Oak

Initially, with reads assigned to the diploid assembly as reference, 137 mutations were called in the upper branch and 44 mutations in the lower branch. From reads assigned to the haploid assembly as reference, 53 mutations were called in the upper branch and 21 mutations in the lower branch. We then visualized the results in the Integrative Genomics Viewer (IGV) for inspection (Robinson *et al.* 2017), including the separate BAM files for short reads from both branches, the original BAM file of short reads from both branches, and the BAM file of HiFi reads used to generate the assemblies. IGV screenshots of both retained and SNVs removed with our manual filters are shown in Supplementary Fig. 11 (available on Figshare).

After IGV inspection, we retained 123 mutations in the upper branch and 14 in the lower branch with the diploid assembly as reference, and 51 mutations in the upper branch and 11 in the lower branch with the haploid assembly as reference. Combining the 2 datasets resulted in 173 mutations in the upper branch and 25 in the lower branch. Because we had called mutations in the upper branch using the lower branch as control and vice versa, there was by definition no overlap between the 2 branches, resulting in a total of 198 SNVs that were absent from the germline (Supplementary Table 9).

All of the 10 upper-branch variants that had been previously validated by amplicon sequencing (Schmid-Siegert *et al.* 2017) were identified with our approach: 2 were detected with the diploid assembly as reference, and 8 were detected with the haploid assembly as reference (Supplementary Table 10). As a control, we called SNVs using Strelka2 (Kim *et al.* 2018), a tool specifically developed for rare frequency somatic mutation. Of the 10 validated upper-branch SNVs (Schmid-Siegert *et al.* 2017), Strelka2 identified 9 SNVs with the haploid assembly as reference. One SNV could not be detected because its region of origin is absent from the haploid assembly. With the diploid assembly as reference, Strelka2 identified 7 of the 10 validated SNVs. Inspecting the alignments of the 3 missing SNVs in IGV indicates that failure to detect them with Streka2 is likely due to the MAPQ values of the corresponding reads being 0 (Supplementary Fig. 12).

For the 7 lower-branch variants confirmed by amplicon sequencing, we did find rare variant reads for these sites also in the upper branch, both in the original and filtered BAM files. Therefore, we do not consider these 7 variants to be true lower-branch-specific mutations compared to the upper branch.

Consistent with mutation spectra observed in other systems (Hofmeister *et al.* 2017; Exposito-Alonso *et al.* 2018; Weng *et al.* 2019; Belfield *et al.* 2021; Satake *et al.* 2023), we found that G:C > A:T mutations were predominant (Fig. 3b).

## Discussion

What does our work say about germline mutation rates? To approximate the per-site germline mutation rates in the Napoleon Oak, we considered only mutations that could be transmitted to the next generation, meaning they should have occurred in L2. As laid out in the Results, we reasoned that L2 accounts for around 60% of the total DNA in leaves. Each haploid set of chromosomes would thus contribute around 30% of the leaf DNA. To identify likely L2 mutations, we therefore used a VAF threshold of at least 0.2 for the haploid assembly as reference and 0.4 for the diploid assembly as reference. We found 77 such potential L2 mutations. For the Napoleon Oak, this would yield a raw mutation rate of approximately 6 × 10^−8^ bp^−1^. Scaled to the age of the Napoleon Oak, 234 years, the annual mutation rate would be approximately 2.5 × 10^−10^ bp^−1^, within the range of rate estimates for other tree species (see the summary in Johannes 2024). We do not address false-negative rates, but even if we accepted all of our 198 originally called mutations to have been fixed in L2 of the sampled leaves, the mutation rate estimate would still be within the range published for other trees.

For acorns produced by the Napoleon Oak today, which was 234 years old at the time of sampling (Schmid-Siegert *et al.* 2017), this would also be close to the generational mutation rate, since we are mostly ignoring mutations that occur only after the entire branch was formed. Assuming that most oak trees have, however, only a generation time closer to 50 years, the generational mutation rate would have about 1.2 × 10^−8^ bp^−1^ as a lower bound. This is not very different from the per-generation mutation rate of *A. thaliana* in nature, estimated to be about 3 × 10^−9^ bp^−1^ (Exposito-Alonso *et al.* 2018).

To assess the per-generation mutation rate, it is also useful to consider the number of cell divisions separating each generation. In the annual species *A. thaliana*, it has been estimated that there are around 30 such cell divisions (Hoffman *et al.* 2004; Watson *et al.* 2016). In trees, there are perhaps twice to thrice as many cell divisions separating each generation (Burian *et al.* 2016). Thus, the per-site per-cell division mutation rate in oak and *A. thaliana* would be very similar. Note that we focus on mutations that are likely to be transmitted to the next generation, and that our study therefore does not speak to whether or not an allometric scaling law needs to be invoked for somatic mutation rates in plants (Johannes 2024).

Recent studies have suggested that mutation rate is affected by a series of biological and environmental factors (Belfield *et al.* 2021). Notably, effective population size has been reported in 2 independent studies to correlate negatively with mutation rate (Sung *et al.* 2012; Lynch *et al.* 2016). In contrast, findings on the effects of generation time vary between taxa: while 1 meta-analysis across eukaryotes reports a positive correlation between generation time and mutation rate, a plant-specific study found a negative correlation (Wang and Obbard 2023; Johannes 2024). This discrepancy may stem from the absence of a segregated germline in plants. *Q. robur* has a long generation time, which may increase the effective germline mutation rate. On the other hand, the large effective population size of oaks may have an opposite effect (Kremer and Hipp 2020). How these factors together influence mutation rate in oaks and other long-lived plants warrants further investigation, using different species and individuals of different ages.

## Supplementary Material

jkaf143_Supplementary_Data

## Data Availability

PacBio HiFi sequencing data have been submitted to the ENA under the project accession PRJEB85730. Assemblies in fasta and gfa format and Supplementary Fig. 11 are available through https://doi.org/10.6084/m9.figshare.28397981. Scripts are available at https://github.com/Wenfei-Xian/Somatic_mutation_in_high_heterogyzous_genome. Supplemental material available at *G3* online.
