# Supplementary material for: Minimizing detection bias of somatic mutations in a highly heterozygous oak genome
Source: G3 (Bethesda). 2025 Jun 21;15(8):jkaf143. doi: 10.1093/g3journal/jkaf143 (PMC12341919; doi:10.1093/g3journal/jkaf143)
Supplement: jkaf143_Supplementary_Data [file jkaf143_supplementary_data.zip › Supplementary_Figures_G3-2025-405931.pdf]

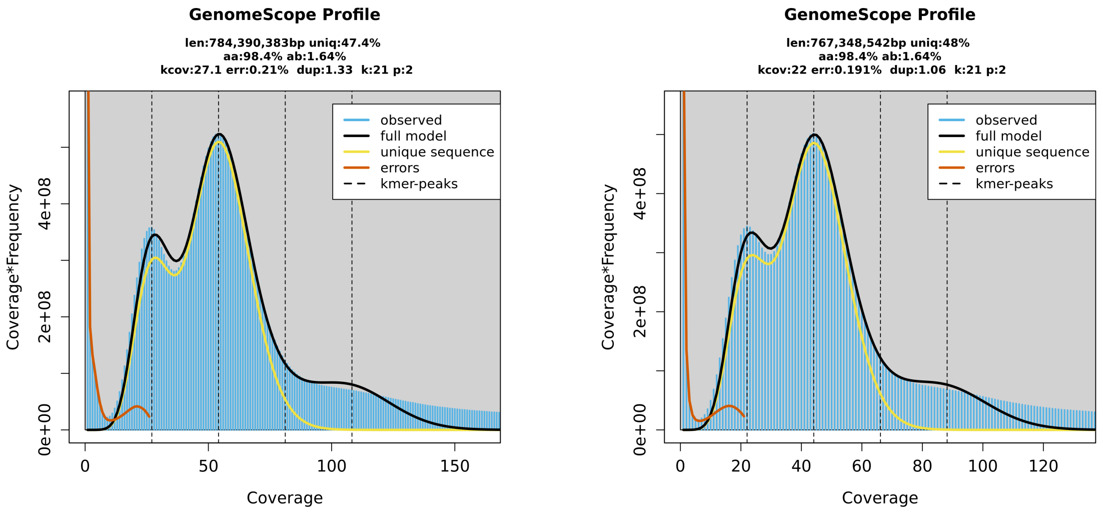

Figure S1. Genome survey using GenomeScope2. A. using illumina reads from lower branch. B. using illumina reads from upper branch.

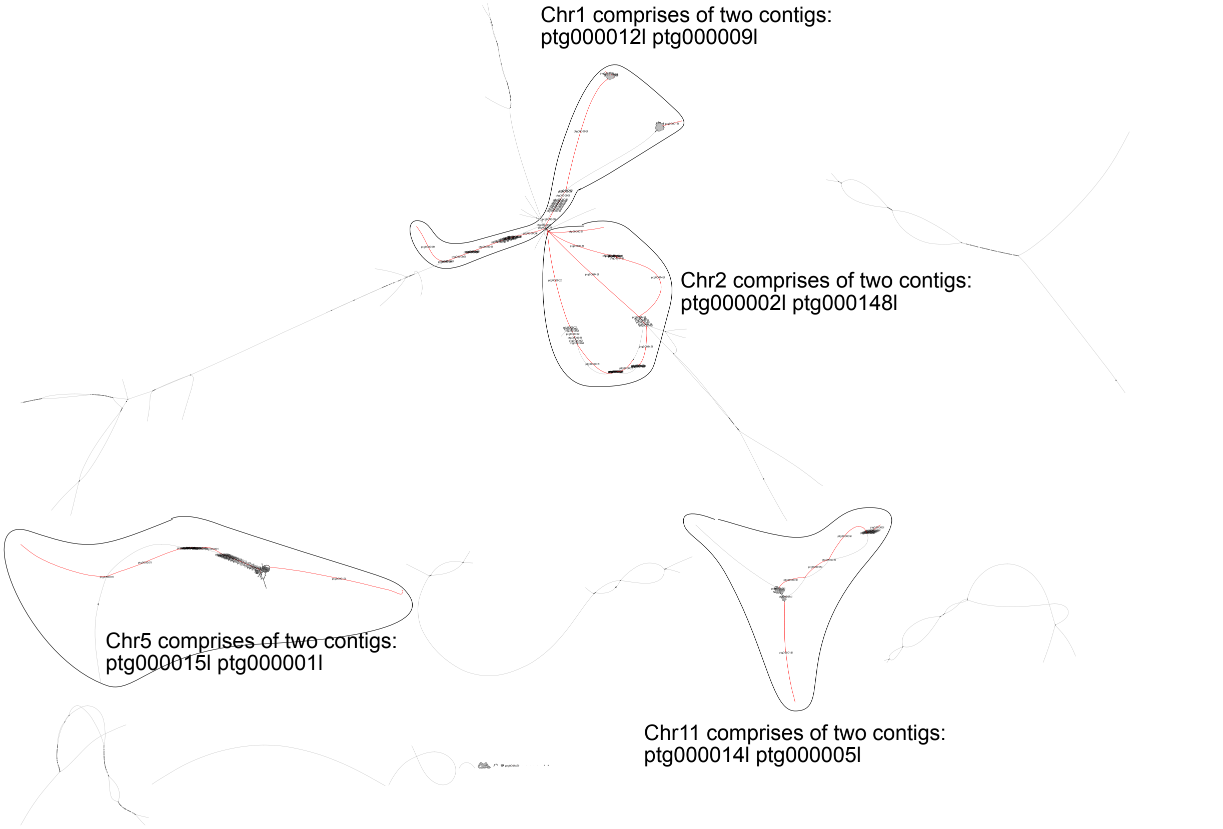

Figure S2. Eight long contigs (in red) lacking two telomeric repeats in the diploid assembly graph for manually scaffolding.

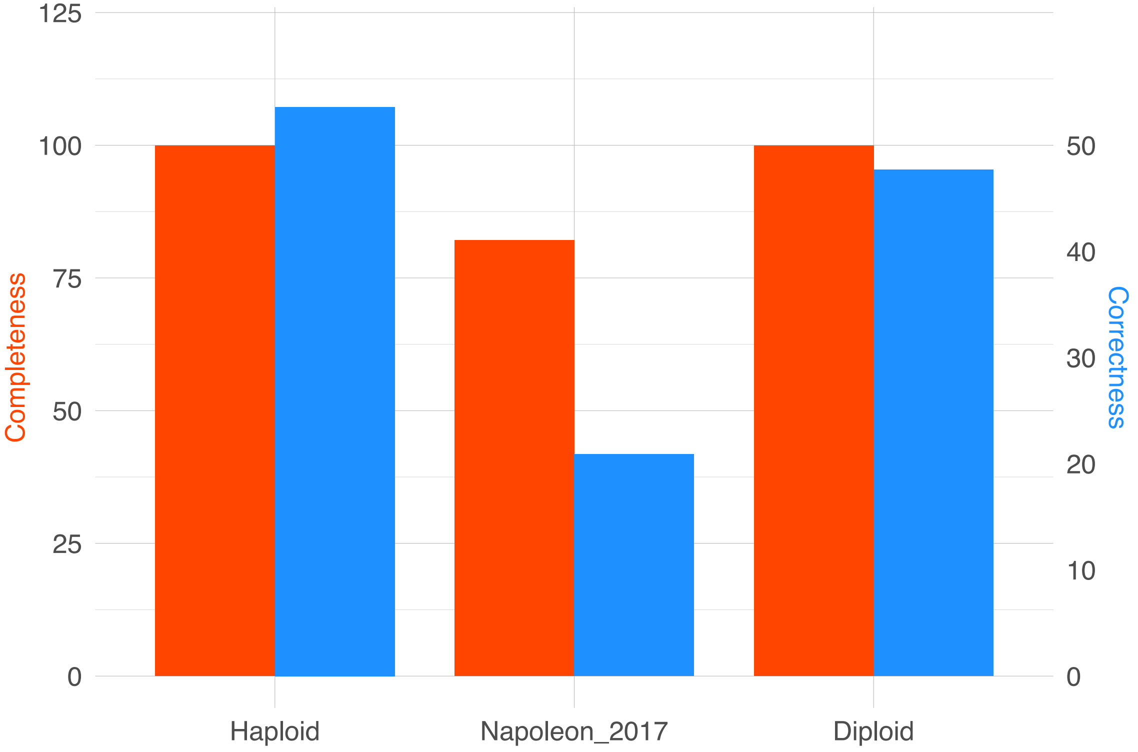

Figure S3. Quality evaluation of two assemblies.

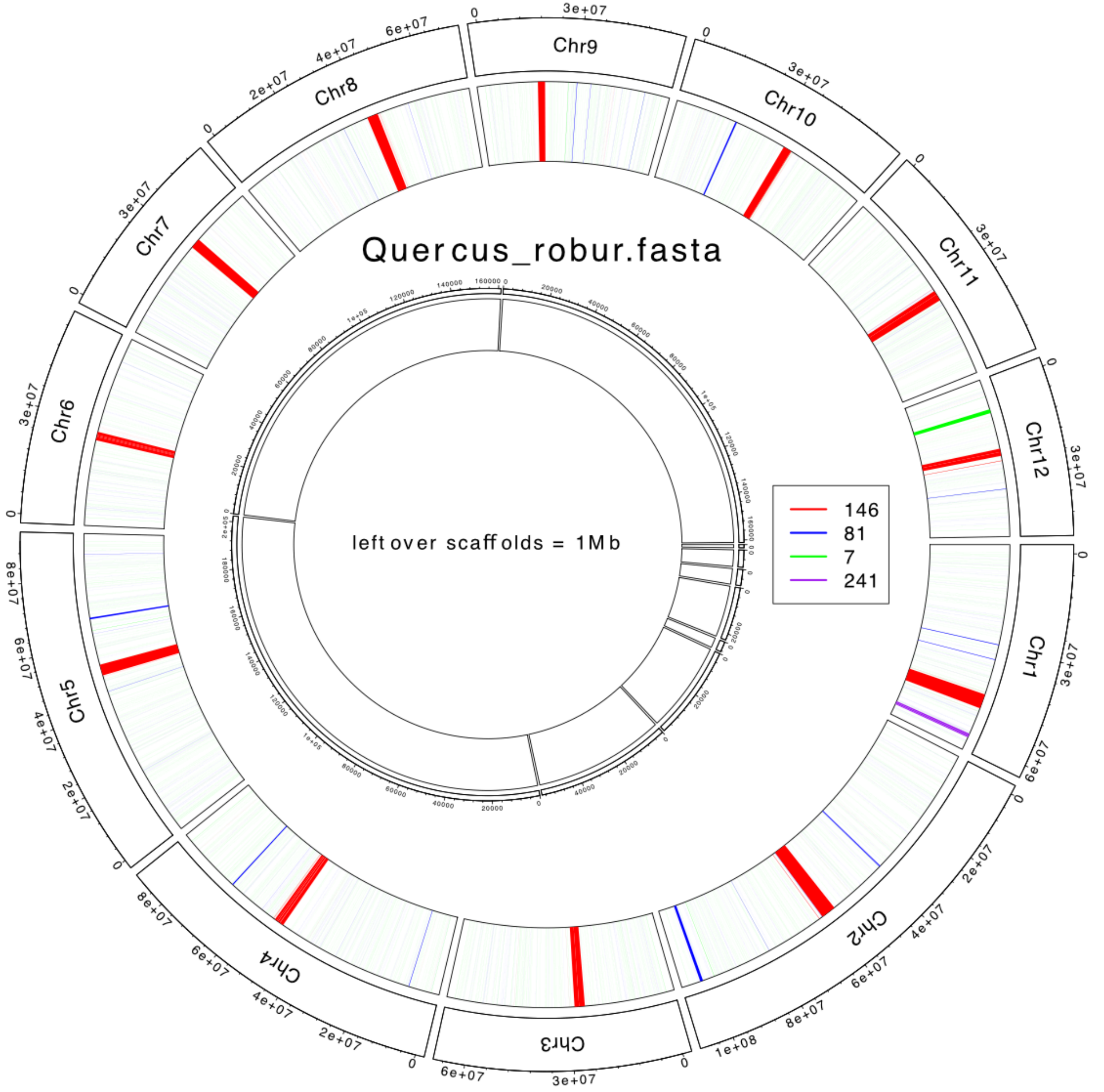

Figure S4. Satellite repeats in haploid assembly.

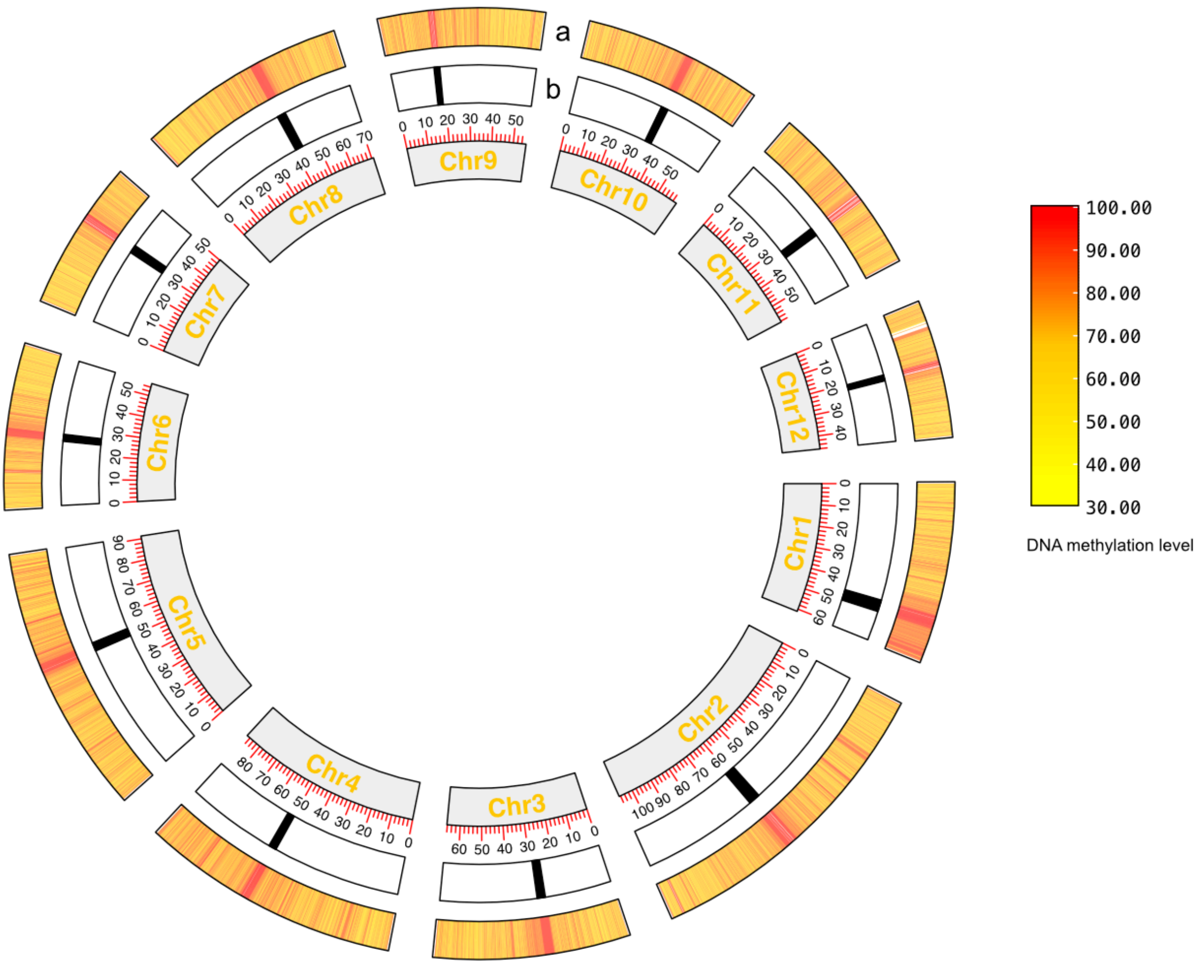

Figure S5. Circos plot of CEN146 and DNA methylation. a track indicates the DNA methylation level and b track indicates the location of CEN146.

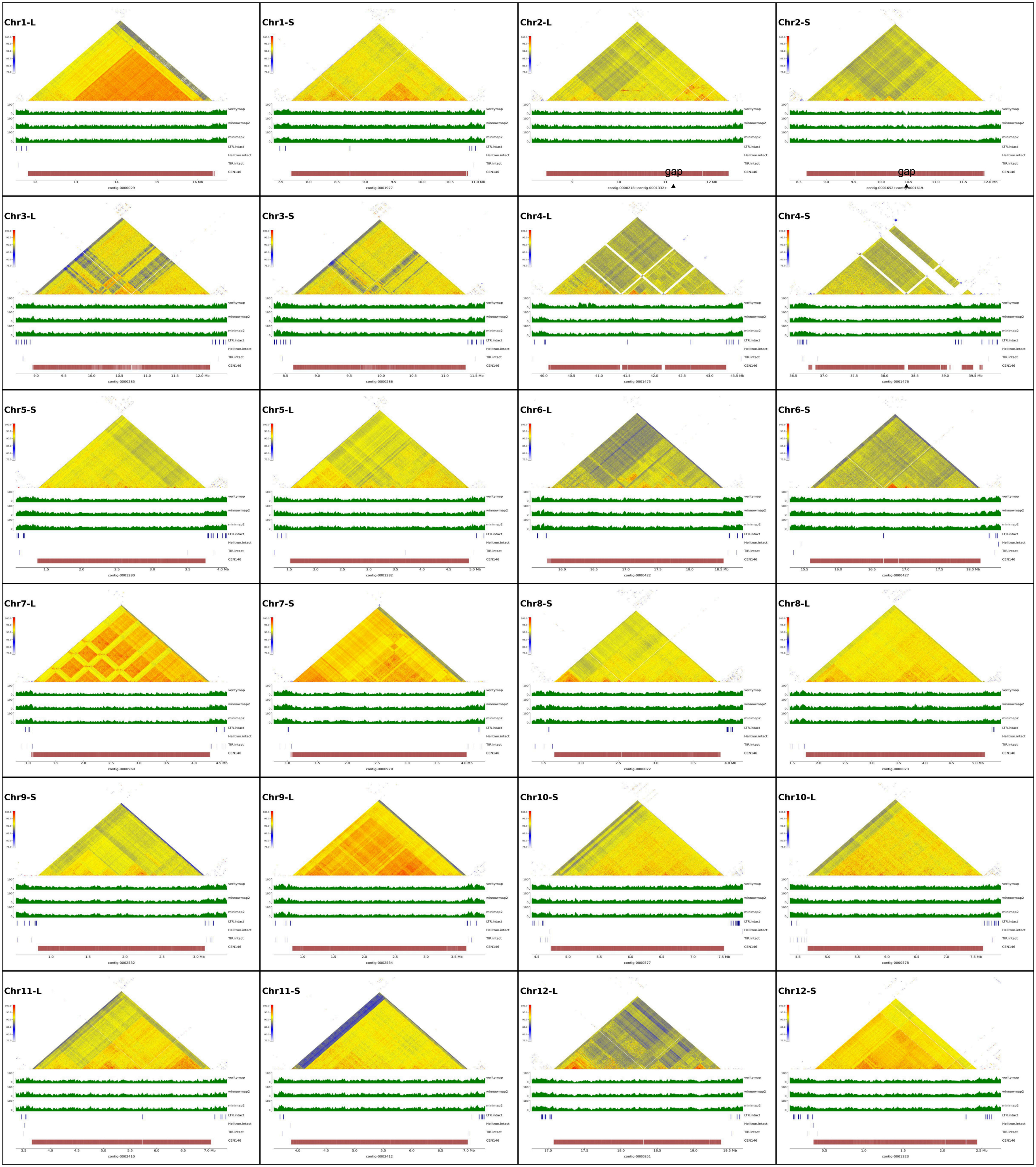

Figure S6. HiFi coverage, the present of intact TE and similarity of CEN146 arrays.

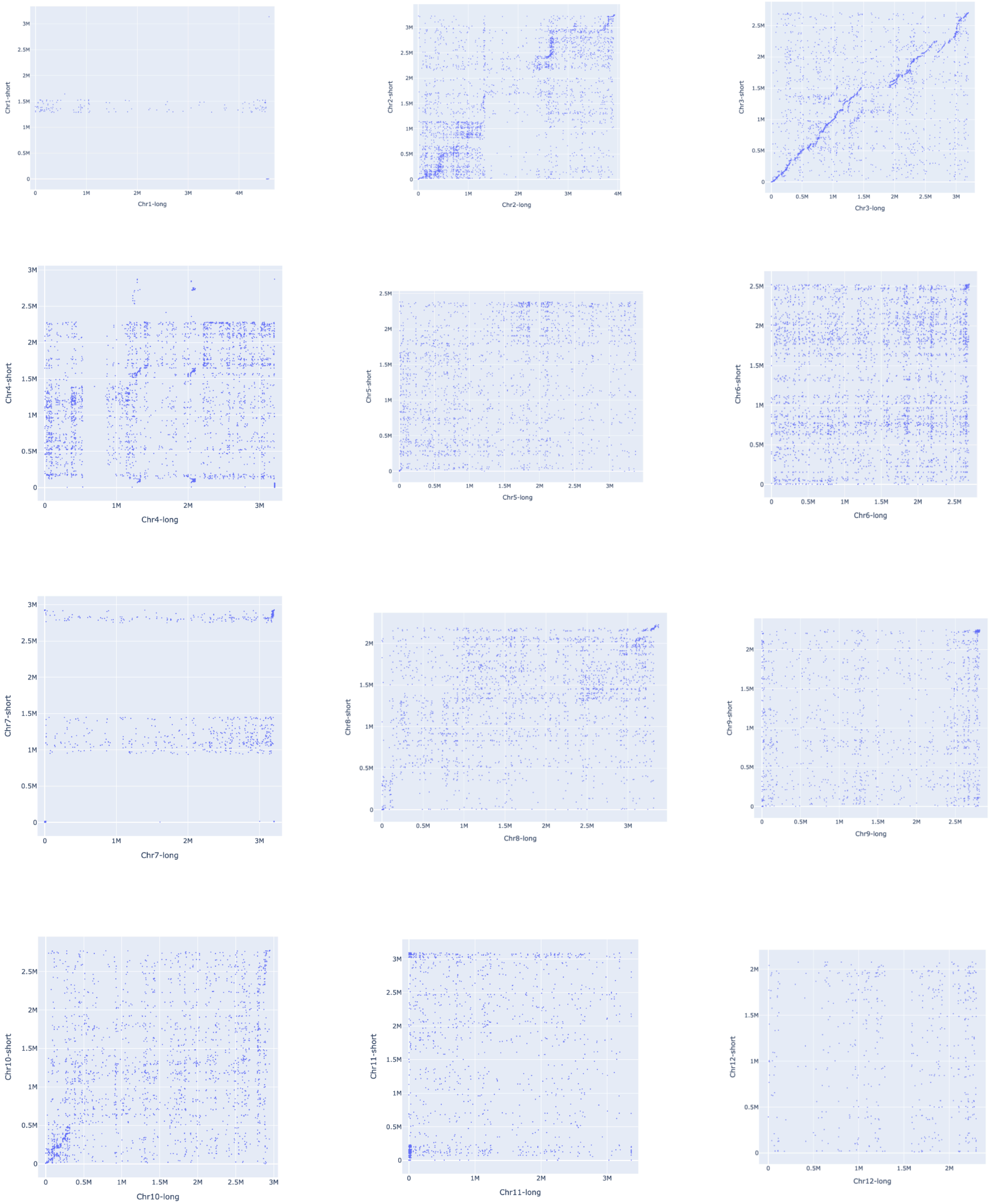

Figure S7. Rare dot-plot of CEN146 arrays between homologous chromosome generated by Unialigner.

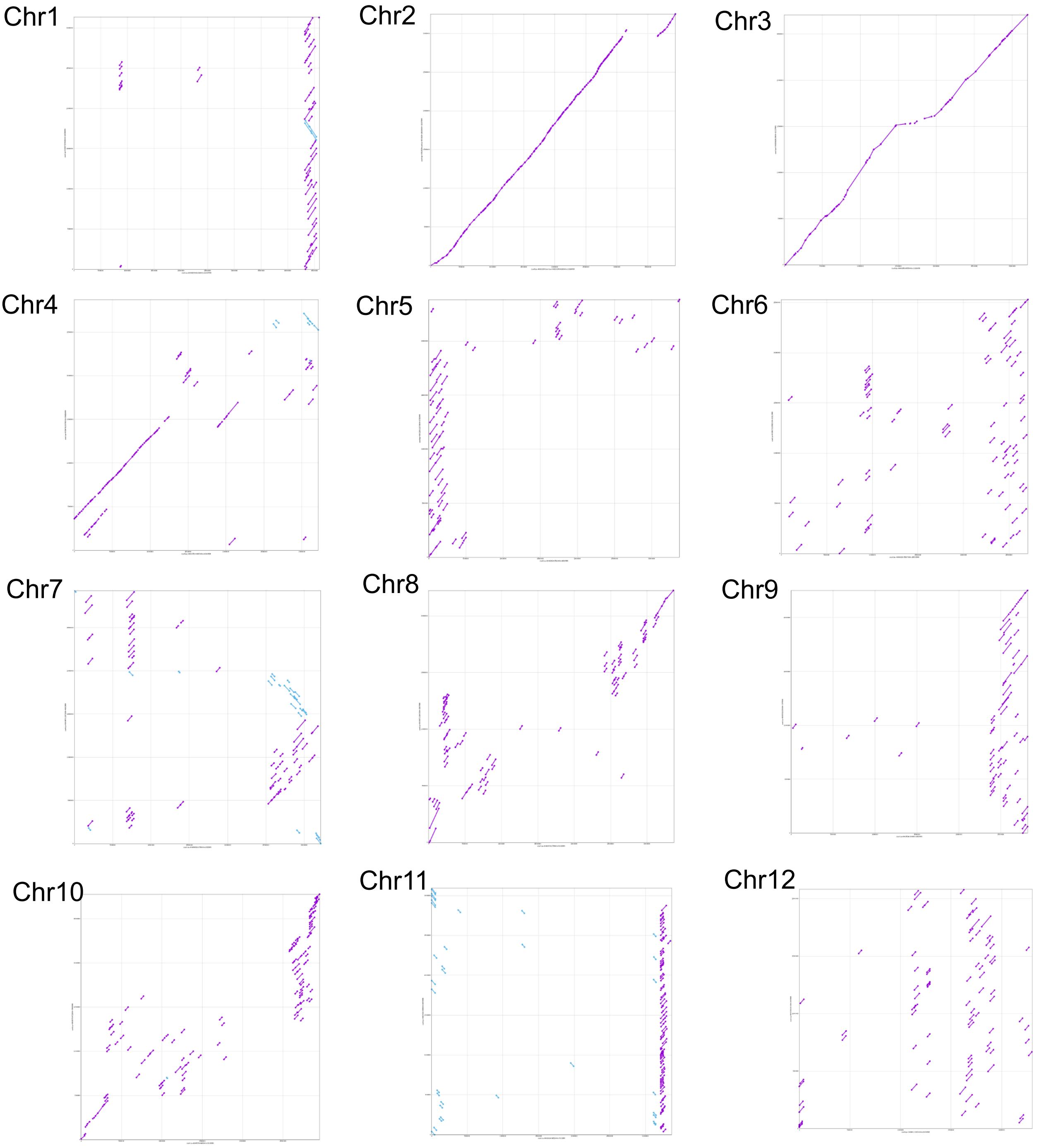

Figure S8. Dot-plot of CEN146 arrays between homologous chromosome by Minimap2.

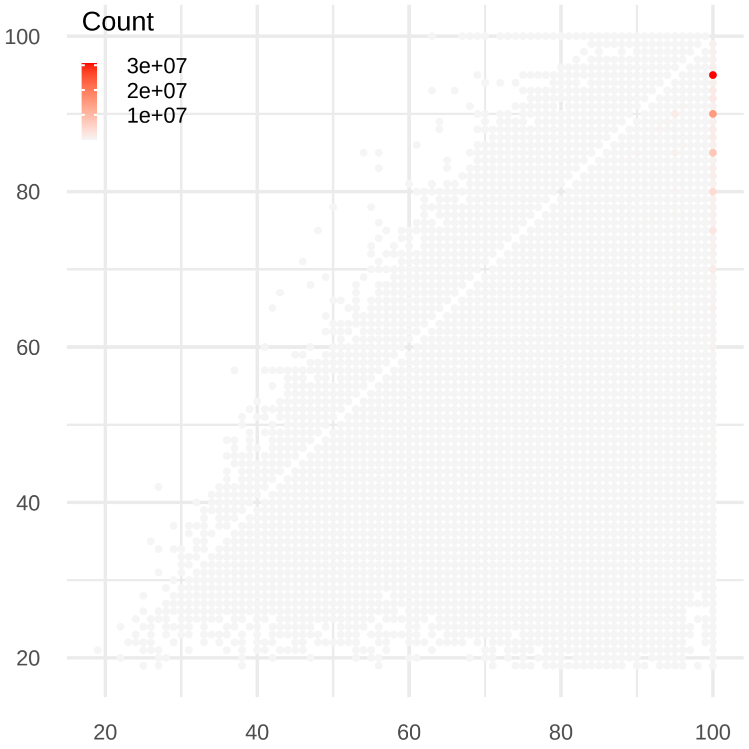

Figure S9. Distribution of alignment score in two assemblies as reference. The color intensity of the dots represent the actual number of reads.

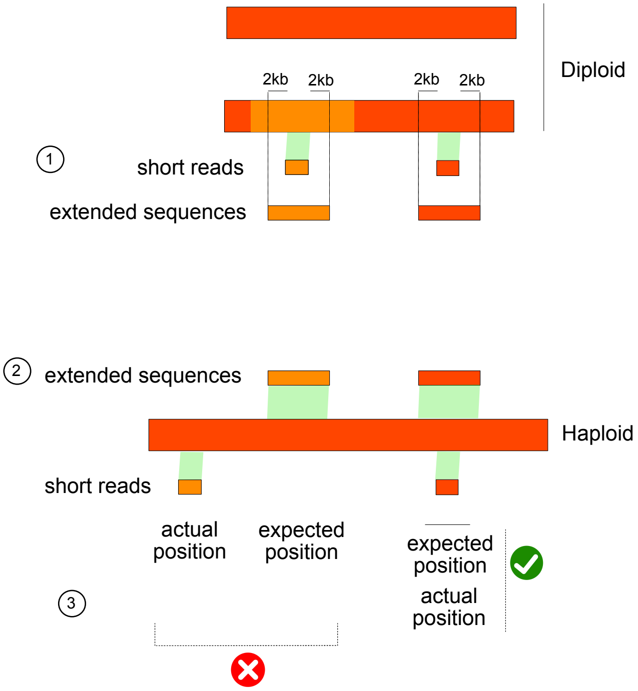

Figure S10. Identification of misaligned short reads in the haploid assembly. Step 1: Align short reads to the diploid assembly and extract the flanking 2 kb sequences. Step 2: Align the short reads and the corresponding extended sequences to the haploid assembly. Step 3: Compare the positions of the short reads and the extended sequences.

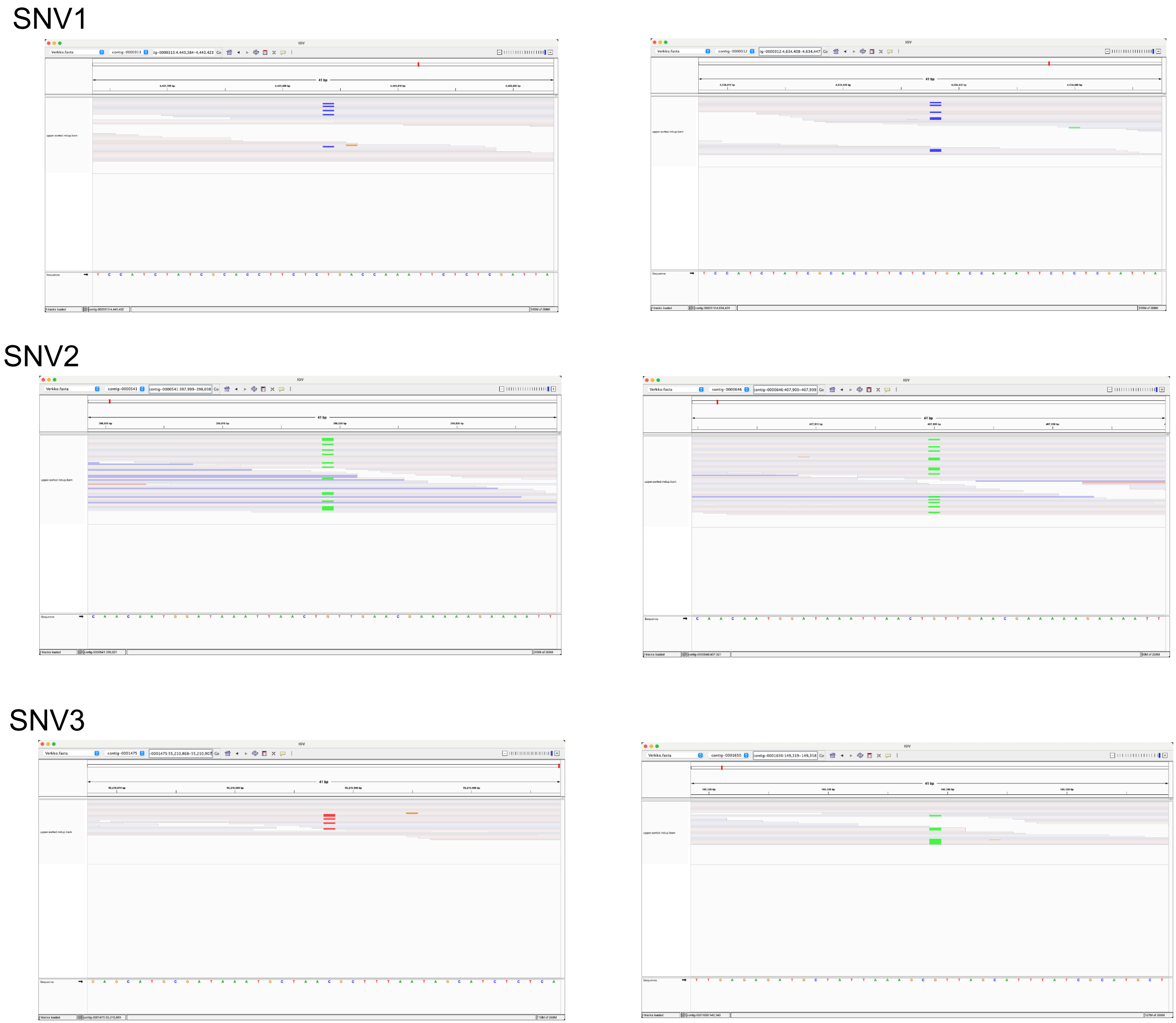

Figure S12. Read alignments for the three undetected SNVs using the diploid assembly as the reference.

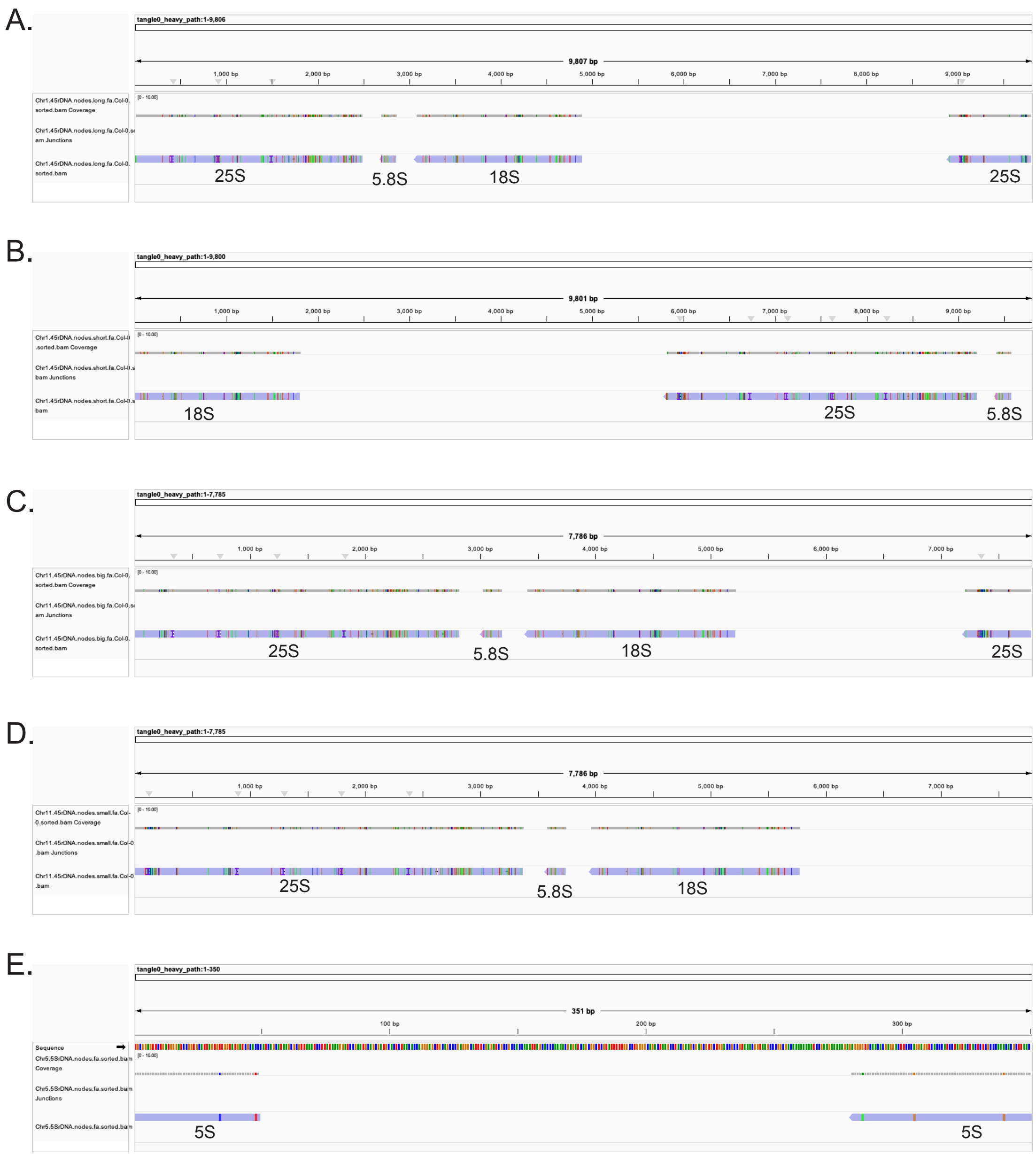

Figure S13. Alignment of *Arabidopsis thaliana* rDNA unit to the oak consensus sequence of each rDNA cluster with BWA. A. 45S rDNA on Chr1 (haplotype 1). B. 45S rDNA on Chr1 (haplotype 2). C. 45S rDNA on Chr11 (haplotype 1). D. 45S rDNA on Chr11 (haplotype 2). E. 5S rDNA on Chr5.

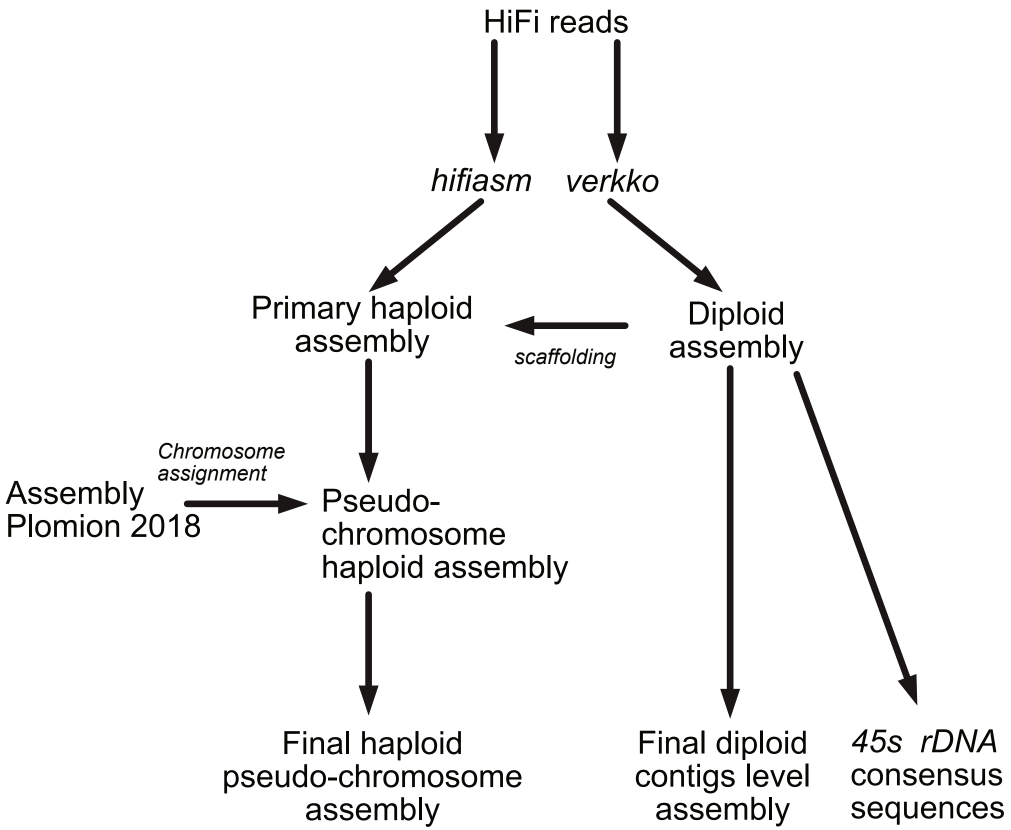

Figure S14. A flow diagram of the nuclear genome assembly steps.
